# Editorial: Emerging mechanistic insights of selective and Nonselective Autophagy in liver and gut diseases and their treatment strategies in the era of COVID-19

**DOI:** 10.3389/fphar.2023.1206291

**Published:** 2023-04-27

**Authors:** Md. Abdul Alim Al-Bari, Manoj B. Menon, Nabil Eid

**Affiliations:** ^1^ Department of Pharmacy, Faculty of Science, University of Rajshahi, Rajshahi, Bangladesh; ^2^ Kusuma School of Biological Sciences, Indian Institute of Technology Delhi, New Delhi, India; ^3^ Department of Anatomy, Division of Human Biology, School of Medicine, International Medical University, Kuala Lumpur, Malaysia

**Keywords:** autophagy, lipophagy, mitophagy, liver diseases, COVID-19, xenophagy

Macroautophagy (hereafter called autophagy), a highly programmed process maintains cellular homeostasis by degrading over-copious or damaged organelles, large macromolecular aggregates, and invading pathogens via the lysosomal system. Autophagy is generally activated by a variety of extracellular or intracellular stresses, such as oxygen- or energy-deprivation, drugs, infections, etc. In addition to non-selective bulk degradation of cytosolic contents, autophagy selectively recycles specific organelles including mitochondria (mitophagy), endoplasmic reticulum (ER) (ER-phagy), lysosomes (lysosomophagy) and intracellular lipid droplets (lipophagy) (Eid. et al., 2013; Klionsky et al., 2021). Autophagy plays several important roles in the regulation of normal liver function and its dysregulation is associated with a wide variety of common and high-risk liver diseases such as metabolic associated fatty liver disease (MAFLD, previously called non-alcoholic fatty liver disease, NAFLD), alcoholic fatty liver disease (AFLD), hepatocellular carcinoma (HCC), and drug-induced liver injury (DILI) as well as their co-existing diseases such as obesity and type 2 diabetes mellitus (T2DM). Xenophagy, another type of selective autophagy which specifically targets intracellular pathogens include virophagy, a process being implicated in coronavirus disease 2019 (COVID-19)-associated hepatic pathologies (Liu et al., 2022). The focus of this Research Topic is to highlight the up-to-date mechanisms of selective and non-selective autophagy in liver and gut diseases. It complies one review article and three research articles.

In a detailed review presented here, Alim Al-Bari et al. describe the mechanistic intricacies of selective autophagy and its impact on liver physiology and pathology and suggest modulation of selective autophagy as key to therapeutic interventions against several hepatic diseases.

In the first research article; Qi et al. report how curcumol, an active constituent of the *Rhizoma Curcumae* roots control AFLD development by targeting hepatocyte senescence. They found that curcumol suppresses lipid accumulation in an ethanol liquid diet-induced liver injury mouse model and in ethanol-treated LO2, human fetal hepatocyte cell line via inhibition of hepatocyte senescence. Mechanistically, the senescence-related suppressive activity of curcumol in AFLD is associated with inhibition of cytoplasmic chromatin fragments (CCF) formation and subsequent inactivation of the cyclic GMP–AMP synthase (cGAS) stimulator of interferon gene (STING) signaling pathway, resulting in a significant reduction in senescence-associated secretory phenotype (SASP) in hepatocytes. The authors concluded anti-AFLD activity of curcumol might be attributed to its blocking of LC3B-lamin B1 interaction for inhibition of CCF formation and subsequent inactivation of the CCF-cGAS-STING signaling pathway, and curcumol is proposed as a therapeutic molecule for hepatocyte senescence.

In developed countries acetaminophen (APAP or paracetamol)-induced liver injury (AILI) is the predominant cause of acute liver failure (Lee, 2017). In their article Yan et al. report that cajaninstilbene acid (CSA), a natural stilbene compound derived from the leaves of pigeon pea [Cajanus cajan (L.) Millsp.] commonly used in traditional medicine alleviates AILI pathology in a mouse model. Since the emergence of the COVID-19 pandemic, it is common clinical practice to advise patients to take APAP to relieve headache and fever (Bertolini et al., 2020). Although APAP is very safe at therapeutic doses, overdose can lead to hepatotoxicity, AILI and even acute liver failure. In this report, the authors show that CSA protects against AILI through enhancing mitochondrial quality control including mitochondrial biogenesis and mitophagy and inhibiting hepatocyte inflammation in response to APAP overdose via Sestrin2-LKB1-AMP-activated protein kinase (AMPK) signaling pathway. From a clinical standpoint, CSA would be a promising drug for treating APAP-induced liver injury. Interestingly, another stilbene analogue resveratrol also protects against APAP-induced liver and kidney injury [Dallak et al., 2022).

MAFLD is the fatty-liver pathology associated with metabolic disorders like obesity and T2DM. The manuscript by Li et al. investigates this cross-talk and describe a beneficial effect of the FDA-approved anti-T2DM drug empagliflozin in ameliorating hepatic steatosis. Empagliflozin is a sodium-glucose co-transporter-1/2 (SGLT1 and 2) inhibitor and synthetic analog of the natural flavonoid phlorizin, isolated from apple trees (*Malus domestica*), rose hips (*Rosa canina* L.), strawberry fruits (*Fragaria x ananassa* Duch.), or pear tree (*Pyrus communis* L.) (Moradi-Marjaneh et al., 2019). In the article by Li et al.s empagliflozin alleviates liver steatosis in db/db mice (8 weeks) and reduces triglyceride content and lipid accumulation in the hepatocyte steatosis model via activation of autophagy through the AMPK-Ten-Eleven Translocation-2 (TET2) signaling pathway. Interestingly, previous study in a rat model of T2DM, empagliflozin was shown to prevent hepatic steatosis, via proposed mechanisms involving activation of SIRT1 and AMPK pathways (Kim et al., 2019). In addition, evidence from the E-LIFT randomized clinical trial indicates that empagliflozin improves MAFLD coexisting with T2DM via reduction of liver fat and serum ALT levels (Kuchay et al., 2018). Based on these findings and previous clinical evidence, empagliflozin can be of great potential in improving outcomes for MAFLD patients with T2DM.

In conclusion, the review article by Alim Al-Bari et al. on this Research Topic summarizes the mechanistic details of selective autophagy in the context of liver and its impact on abnormal liver function in patients including that of COVID-19. In addition, this review proposes the pharmacological targeting of autophagy as a viable strategy against liver pathologies, which is supported by the findings of the three research articles in the Research Topic. While modulation of autophagy seems to be a sought-after strategy for treating several diseases, focus on selective autophagy pathways and development of specific inhibitors for the same will be the way forward. We hope the research articles and the comprehensive review on this Research Topic presented here generate more interest in research aimed at understanding molecular mechanisms of autophagy-related liver pathologies, finally providing reasonable clinical treatment options.
